# Supplementary material for: SPARC Is Highly Expressed in Young Skin and Promotes Extracellular Matrix Integrity in Fibroblasts via the TGF-β Signaling Pathway
Source: Int J Mol Sci. 2023 Jul 29;24(15):12179. doi: 10.3390/ijms241512179 (PMC10419001; doi:10.3390/ijms241512179)
Supplement: Supplementary file 1 [file ijms-24-12179-s001.zip › ijms-2466791-supplementary.pdf]

Supplementary materials

SPARC is highly expressed in young skin and promotes extracellular matrix integrity in fibroblasts via the TGF-β signaling pathway

Seung Min Ham [1], Min Ji Song [2,3,4], Hyun-Sun Yoon [2,3,4,5], Dong Hun Lee [2,3,4], Jin Ho Chung [2,3,4,6], and Seung-Taek Lee [1,*]

[1] Department of Biochemistry, College of Life Science and Biotechnology, Yonsei University, Seoul 03722, Korea; mongsil1115@naver.com (S.M.H)
[2] Department of Dermatology, Seoul National University College of Medicine, Seoul 03080, Korea; minjisong@snu.ac.kr (M.J.S); hsyoon79@gmail.com (H.-S.Y); ivymed27@snu.ac.kr (D.H.L); jhchung@snu.ac.kr (J.H.C)
[3] Laboratory of Cutaneous Aging Research, Biomedical Research Institute, Seoul National University Hospital, Seoul 03080, Korea
[4] Institute of Human-Environment Interface Biology, Seoul National University, Seoul 03080, Korea
[5] Department of Dermatology, Seoul National University Boramae Hospital, Seoul 07061, Korea
[6] Institute on Aging, Seoul National University, Seoul 03080, Korea

[*] Correspondence: stlee@yonsei.ac.kr; Tel.: +82-2-2123-2703

Supplementary Table S1. Primer sequences for RT-PCR of human SPARC and nine selected DEGs.

Gene Symbol	Nucleotide sequence (5′ → 3′)	Nucleotide position	Annealing temp (℃)	GenBank No.
SPARC	AGACCATGAGTATGTGTAACAGGAGG<br>CTTGAAATGTTGCTAGTGTGATTGG	2616-2641<br>2771-2747	59.5	NM_003118
ELN	ACCTGGTTGACCTGTCATGGC<br>ACTGCTCTGAAGTTCAGTGGACC	2916–2936<br>3094–3072	56.3	NM_000501
TSPAN2	GTCCTCTGCTGTGCGATACGA<br>GCATTCACTCAAGGGGTAAACCAG	696–716<br>916–893	61.3	NM_005725
COMP	ACGTGGTCTTGGACACAACCAT<br>AGCTGATGGGTCTCATAGTCCTCT	2165–2186<br>2297–2275	61.3	NM_000095
OLFM2	AGTATTCCCACATCTCGATGCTGG<br>GATCACCCTTGAGGGACACAGG	1387–1410<br>1601–1580	61.3	NM_058164
WNT2	TTCTAGAGCAGTTGGCCAAGCA<br>TACCCCTAAGGGTGGTAGCTGT	1479–1500<br>1692–1671	61.3	NM_003391
CCN2	CCCCAGTGACAGCTAGGATGT<br>TGCCACAAGCTGTCCAGTCT	1643–1663<br>1798–1779	56.3	NM_001901
TSPAN13	TGGCTGACCTACAGATACAGGAAC<br>TGCCACCGAGCTTCAGAGAC	766–789<br>1047–1028	61.3	NM_014399
SERPINE1	CAGACCTGGTTCCCACTGAGG<br>GCCAGTGCCACAGTGGACT	1679–1699<br>1880–1862	61.3	NM_000602
PTGS2	CACTGCAGGCCTGGTACTCA<br>CATGCAGGTAGCCAGGCTTGA	2939–2958<br>3188–3168	61.3	NM_000963

Supplementary Table S2. Reads per kilobase million (RPKM) values for the selected 9 DEGs and human *COL1A1*, *MMP-1*, and *GAPDH* genes.

Gene Symbol	Con	SPARC	$\log_2$(fold change)	*p* value	*q* value
COL1A1	1,876.17	3,450.10	0.97	1.1×10^{-5}	2.2×10^{-3}
ELN	23.42	186.26	3.07	1.5×10^{-37}	3.4×10^{-33}
TSPAN2	0.95	9.67	2.72	3.9×10^{-19}	1.2×10^{-15}
COMP	8.06	38.80	2.33	2.0×10^{-21}	8.5×10^{-18}
OLFM2	14.65	62.86	2.19	3.3×10^{-19}	1.2×10^{-15}
WNT2	13.65	49.74	2.02	2.3×10^{-17}	5.5×10^{-14}
CCN2	39.97	120.37	1.65	5.0×10^{-13}	6.9×10^{-10}
TSPAN13	6.05	16.52	1.51	4.2×10^{-9}	2.4×10^{-6}
SERPINE1	249.93	680.20	1.51	2.1×10^{-11}	2.3×10^{-8}
MMP-1	3,167.69	1,099.07	-1.46	6.7×10^{-11}	6.3×10^{-8}
PTGS2	23.50	7.06	-1.78	9.8×10^{-14}	1.5×10^{-10}
GAPDH	3,729.40	3,395.69	-0.07	7.4×10^{-1}	1.0

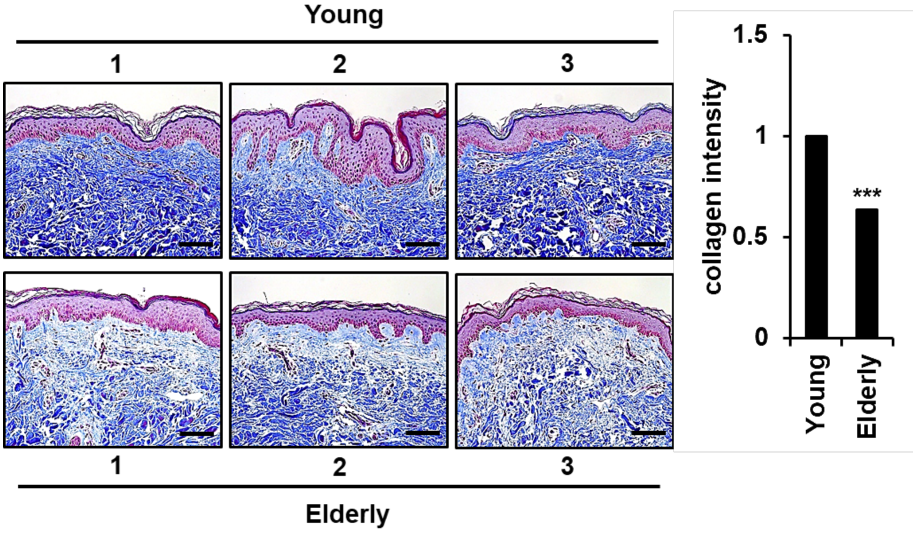

Supplementary Fig. S1. Analysis of collagen intensity in human skin tissues.
Formalin-fixed and paraffin-embedded sections of human skin tissues were deparaffinized and subjected to Masson's trichrome staining [61]. The collagen intensity [62] was quantified using Image J software (National Institutes of Health, Bethesda, MD, USA) by measuring the staining intensity at three locations in each tissue section. The graph shows a comparison of the relative collagen intensity between elderly human skin tissues and young tissues. Values are shown as means ± SD of three independent experiments. ***$p < 0.001$ *vs*. young tissues. Magnification, ×200. Bar, 100 μm.

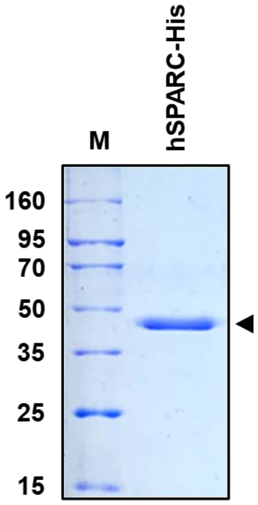

Supplementary Fig. S2. Purification of recombinant human SPARC-His.
His-tagged recombinant human SPARC protein purified from conditioned medium of HEK 293 cells stably transfected with pcDNA3.1-hSPARC-His was resolved by SDS-PAGE and stained with Coomassie brilliant blue. M, molecular weight marker.
